# Widespread Myocardial Delivery of Heart-Derived Stem Cells by Nonocclusive Triple-Vessel Intracoronary Infusion in Porcine Ischemic Cardiomyopathy: Superior Attenuation of Adverse Remodeling Documented by Magnetic Resonance Imaging and Histology

**DOI:** 10.1371/journal.pone.0144523

**Published:** 2016-01-19

**Authors:** Eleni Tseliou, Hideaki Kanazawa, James Dawkins, Romain Gallet, Michelle Kreke, Rachel Smith, Ryan Middleton, Jackelyn Valle, Linda Marbán, Saibal Kar, Rajendra Makkar, Eduardo Marbán

**Affiliations:** 1 Cedars-Sinai Heart Institute, Los Angeles, CA, United States of America; 2 Keio University School of Medicine, Tokyo, Japan; 3 Capricor Inc., Los Angeles, CA, United States of America; Georgia Regents University, UNITED STATES

## Abstract

Single-vessel, intracoronary infusion of stem cells under stop-flow conditions has proven safe but achieves only limited myocardial coverage. Continuous flow intracoronary delivery to one or more coronary vessels may achieve broader coverage for treating cardiomyopathy, but has not been investigated. Using nonocclusive coronary guiding catheters, we infused allogeneic cardiosphere-derived cells (CDCs) either in a single vessel or sequentially in all three coronary arteries in porcine ischemic cardiomyopathy and used magnetic resonance imaging (MRI) to assess structural and physiological outcomes. Vehicle-infused animals served as controls. Single-vessel stop-flow and continuous-flow intracoronary infusion revealed equivalent effects on scar size and function. Sequential infusion into each of the three major coronary vessels under stop-flow or continuous-flow conditions revealed equal efficacy, but less elevation of necrotic biomarkers with continuous-flow delivery. In addition, multi-vessel delivery resulted in enhanced global and regional tissue function compared to a triple-vessel placebo-treated group. The functional benefits after global cell infusion were accompanied histologically by minimal inflammatory cellular infiltration, attenuated regional fibrosis and enhanced vessel density in the heart. Sequential multi-vessel non-occlusive delivery of CDCs is safe and provides enhanced preservation of left ventricular function and structure. The current findings provide preclinical validation of the delivery method currently undergoing clinical testing in the Dilated cardiomYopathy iNtervention With Allogeneic MyocardIally-regenerative Cells (DYNAMIC) trial of CDCs in heart failure patients.

## Introduction

The field of cell therapy has been plagued by uncertainties regarding delivery methods[1], a factor likely contributing to inconsistent results in the clinic [2,3]. More specifically, in cell therapy for myocardial infarction (MI), the standard stop-flow intracoronary (IC) approach uses an over-the-wire angioplasty catheter, which is repeatedly inflated in the infarct-related artery; cells are infused during inflations [4]. However, there is no evidence that vessel occlusion is necessary for efficacy. Moreover, the stop-flow approach may not be well-suited for treating non-focal conditions such as cardiomyopathies, [5,6] where broad myocardial coverage is desirable. Although systematic preclinical optimization of delivery in large-animal models is rarely performed, we posit that such studies are vital as they may decrease the number of failures in translation [7–9].

Based on this premise, we questioned prevailing assumptions regarding the need for stop flow, and we tested multivessel delivery in an effort to achieve safe, effective delivery to the pumping chambers of the heart. We first compared single-vessel stop-flow and continuous single-vessel non-occlusive IC delivery of cells in a porcine model of chronic MI. Allogeneic cardiosphere-derived cells (CDCs), which induce myocardial regeneration when delivered via the stop-flow technique, [10–13] were used as the therapeutic agents to compare efficacy. Second, we tested the safety and efficacy of sequential multi-vessel non-occlusive IC delivery of CDCs, using magnetic resonance imaging (MRI) and histology to assess cardiac structure and function. The use of MRI establishes direct links to clinically-tractable *in vivo* endpoints, while the use of histology provides validation for the MRI findings as well as useful information regarding the effects of cell therapy on angiogenesis and fibrosis.

## Methods

All animal procedures were conducted in accordance with humane animal care standards outlined in the NIH Guide for the Care and Use of Experimental Animals. The experiments were approved by the Cedars-Sinai Medical Center Animal Care and Use Committee (IACUC, # 3661). The single-vessel and three-vessel studies were performed contemporaneously. Female Yucatan mini pigs (40–45 kg) underwent 150 min occlusion of the mid-left anterior descending (LAD) artery, three to four weeks later were assigned either to single-vessel or multi-vessel protocols (depicted schematically in S1 Fig). Contrast-enhanced MRI was performed in both studies 48 hrs before (Baseline) and 4 weeks after infusions (follow-up), after which animals were euthanized. Extensive cell manufacturing experiments and histological analysis to address the mechanisms beyond the functional results were performed in the three vessel study.

### Study design

For single-vessel left anterior descending (LAD) coronary arterial infusion, animals (n = 15) were randomly assigned to IC delivery of 12.5M allogeneic CDCs under stop-flow (n = 5), or continuous-flow infusion of CDCs over 10 min (n = 5). Animals infused with vehicle under continuous flow technique served as controls (n = 5).

For the multi-vessel study, 16 animals were assigned to IC delivery of 12.5M CDCs sequentially in the LAD, the left circumflex (LCX) and the right coronary artery (RCA) either under stop-flow as described above (n = 5), or with continuous flow (n = 6). Animals infused with vehicle under stop-flow served as controls (n = 5; S1 Fig). MRI was performed 48 hrs before infusion (Baseline) and 1 month (follow-up) after infusions. All pigs were euthanized at 4 weeks, just after the endpoint MRI. For the short term engraftment evaluation, n = 3 animals were infused with 12.5M Luc+ CDCs in each of the three coronary vessels under continuous-flow. One animal was excluded due to EF>50%.

### Cell preparation

Allogeneic CDCs were isolated and manufactured from two different male donors as described [10–13]. More specifically, two male donor Sinclair pigs (#0682 and #0111) were euthanized and their hearts were excised under sterile conditions. Both donors express 12–15 allo-mismatched swine leukocyte antigen (SLA) haplotypes with the recipients to ensure allogeneicity [13]. The hearts were dissected into ~25mg pieces which were seeded to create explant derived cells (EDCs). A Master Cell Bank (MCB) was created from EDCs harvested ~14 days later. MCB vials were thawed and cultured as cardiospheres (CSps) on Ultra Low^®^ Cell STACK^®^ vessels (Corning Life Sciences). Allogeneic CDCs were grown by seeding CSps on Nunc Triple Flasks (Thermo Scientific), and passaging when confluent. Allogeneic CDCs were resuspended (1.25M/ mL for a total dose of 12.5M) in CryoStor^™^CS10 (BioLife Solutions) in cryobags (PL07 PermaLife Bags, OriGen Biomedical Inc), placed directly in a CryoMed controlled-rate freezer, and then transferred to liquid nitrogen. The bags were thawed on the day of infusion. Upon thawing, 1 mL of heparin (100 USP units/ mL) and 0.1 mL nitroglycerin (50 μg/ mL) were added as diluents for a total 10 mL volume of administration. Before infusion, cell number and viability were evaluated. Phenotypic characterization of the CDCs before and after freezing was also performed by evaluating the expression of surface markers (CD105 and CD45, BD Pharmingen).

### Cell Infusion

**Fig 1:**
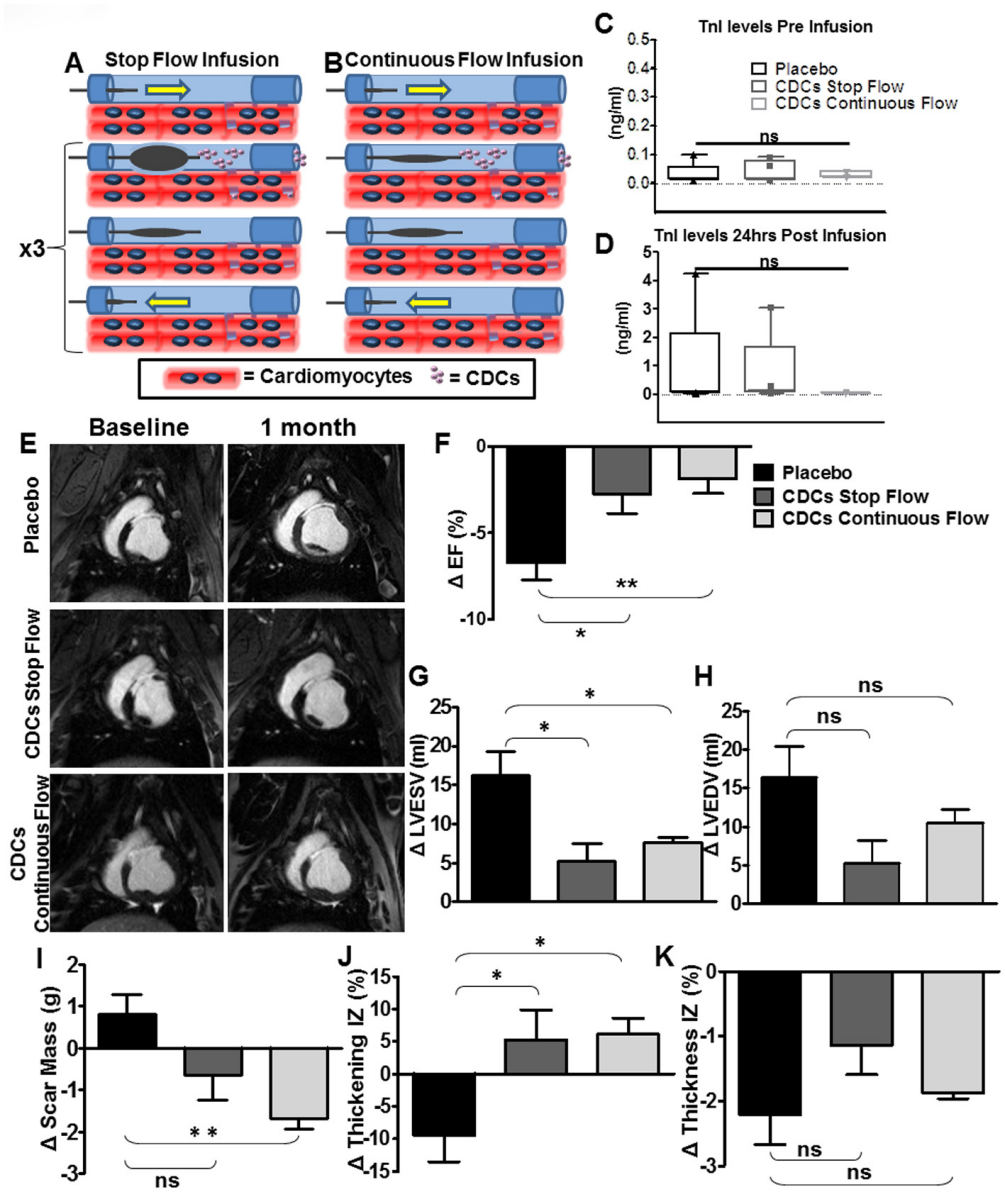
Single-vessel Study. **A**. Illustration of stop-flow protocol and **B**., the continuous-flow protocol. **C, D**. Box Plots showing serum [TnI] before infusion and 24hrs post infusion (p = ns; normal <0.05ng/ml). **E**. Short-axis contrast-enhanced images at baseline and 4 weeks after CDC or vehicle infusion showing **F**. significant changes of ejection fraction in placebo versus either treated group (p = 0.03 placebo vs CDCs stop-flow and p<0.01 placebo vs CDCs continuous-flow). **G**. Changes in end-systolic volume between baseline and 4 weeks post-infusion (p = 0.02 placebo vs CDCs stop-flow and p = 0.05 placebo vs CDCs continuous-flow), and **H**., in end-diastolic volume (p = 0.06 placebo vs CDCs stop-flow and p = 0.2 placebo vs CDCs continuous-flow). Changes in scar mass (**I**, p = 0.08 placebo vs CDCs stop-flow and p<0.01 placebo vs CDCs continuous-flow), **J**., infarct wall motion (p = 0.04 placebo vs CDCs stop-flow and p = 0.02 placebo vs CDCs continuous-flow) and **K**., infarct wall thickness between placebo and treated groups (p = ns). Error bars indicate SEM. * p<0.05, ** p<0.01

Three weeks after MI, animals underwent baseline MRI. Animals with EF<50% were included in the study (one pig was thereby excluded). Through an over-the-wire catheter (TREK^®^, Abbott Vascular), vehicle (CS-10) or CDCs were infused down the LAD. For stop-flow infusion, the balloon was inflated in the LAD segment where we had previously created the MI and cells were infused during three 3-min stop-flow cycles separated by 3 min rest intervals (Fig 1A) under angiographic monitoring. For continuous-flow infusion, the over-the-wire catheter was advanced in the LAD, to the site where we had previously created the MI, and cells or vehicle were infused over a 10 min period without inflating the balloon (Fig 1B). The infusion catheter position was verified angiographically every 5 min.

For multi-vessel infusions under stop-flow, the balloon was inflated first in the LAD and cells infused as described above. Following LAD infusion, the balloon catheter was repositioned to the mid-LCX and cells were infused similar to the LAD. Finally the RCA was infused with the balloon inflated in the mid portion of the vessel. For multi-vessel continuous flow infusions, cells were infused first in the LAD, then the LCX and finally the RCA, mimicking the methods described for single-vessel nonocclusive delivery. Immediately after cell infusion, angiography was repeated to confirm patency and to evaluate coronary flow according to four TIMI grades as described [14].

Blood samples were drawn before and 24 hrs after infusions to evaluate serum levels of troponin I (TnI) as a biomarker of myocardial injury [15].

### Short-Term CDC Engraftment by Luciferase

We assessed engraftment in multivessel continuous-flow pigs (n = 3). In these experiments, CDCs were transduced with an adenoviral vector carrying the firefly luciferase gene (S2A Fig) and pigs were euthanized 24 hours after infusion as described [11] (S2B Fig). The LAD, LCX and RCA territories below the level of the CDC infusions, defined by anatomic marks during the angiography were separately processed and analyzed. All three heart regions were sectioned into one-gram tissue samples and homogenized with 10% fetal bovine serum and tissue lysis buffer followed by centrifugation [11]. Luciferase activity of the supernatant was measured and converted to CDCs/g tissue using standard curves created specifically for each luciferase-transduced batch of CDCs, thereby averting potentially-confounding effects of differences in transduction efficiency (Luciferase assay system, Promega). More specifically, for the standard curve we measured the luciferase signal in known concentrations of CDCs from the same transduced badges of cells used for the *in vivo* infusions after mixing with myocardial tissue derived from the atria (we included a max of 5M cells followed by 2.5M, 1M, 500.000, 250.000, 125.000 cells and a no cell sample; each one mixed with 1gr of tissue). This assay allowed us to validate the activity of the enzyme and the viability of the cells.

### MRI acquisition and analysis

Cardiac MRI was performed ~48hrs before infusion and 4 weeks afterwards. All studies were done with 3-T, Siemens Magnetom Verio (Erlangen, Germany). ECG-gated, breath-hold cine MRI sequences in short axis, vertical axis, and horizontal long axis were used to assess function without including the papillary muscles. For bulk motion evaluation, the LV endocardium and epicardium were manually segmented for each cardiac phase image and for each phase, 100 evenly spaced chords were virtually defined. In short axis, the LV was completely encompassed by contiguous 6-mm-thick slices. Infarct area was defined by applying a region-growing algorithm that observes the full-width half maximum (FWHM) criterion on late-gadolinium-enhanced (LGE) images (i.e., 8–10 min after injection of 0.2 mmol/kg body weight of gadopentate dimeglumine (Magnevist, Bayer Healthcare Pharmaceuticals)). This approach has lower inter-observer bias and superior accuracy of infarct volume compared to other techniques [16]. All MRI studies were analyzed independently on an offline workstation (CVi42, Circle Cardiovascular Imaging Inc, Calgary, Canada) by two investigators unaware of treatment allocation (RG and JD). In the analysis we included global LV function (LV end-diastolic volume [LVEDV] and LV end-systolic volume [LVESV]). Scar mass and viable mass were calculated from LGE images and interpreted using the American Heart Association 17- segment model [17,18].

Regional wall thickening and thickness were also quantified as described [19]. Short axis slices were manually assigned to basal, mid or apical left ventricular slices and divided into 100 equally spaced chords. Based on matched delayed contrast-enhanced images the hyper enhanced zone was defined as the infarcted zone (IZ) and segments 60 degrees away from the infarcted zone were defined as remote zone (RZ). The areas between infarcted and remote zone were identified as border zone (BZ). For each segment, regional function parameters were calculated from end-diastolic and end-systolic contours. Values of thickening and thickness for all single segments were averaged to derive regional function parameters of these zones.

For the multi-vessel study we additionally performed regional analysis based on tagged images using harmonic phase (HARP^®^) software (Diagnosoft; Cary, NC) as described [9]. Two midventricular contiguous short axis tagged images including the scar were included in the analysis. The RV insertion was used as a marker to create a 24-segment mesh based on defined epicardial and endocardial contours. In parallel to the LGE images the tagged images were segmented into IZ, BZ and RZ as described above. The peak Lagrangian strain defined as deformation from its original length was measured by averaging the peak from each individual segment. For mechanical dyssynchrony the circumferential uniformity ratio estimate was evaluated as previously described [20]. Briefly, a mesh to divide each short-axis slide in 24 evenly distributed segments was created and plotted versus circumferential strain. Each plot was then subjected to Fourier analysis. The ratio of mean to mean plus first-order power provided the CURE index.

### Histology

Hearts were harvested after euthanasia in the multi-vessel groups and sectioned in ~8mm thick slices from the apex to the level of the mitral valve and stained with 1% 2,3,5- triphenyltetrazoliumchloride (TTC) for 15 min at 37°C to define the infarct. Both sides of the tissue sections were photographed, image borders were drawn using Image J, (version 1.48, NIH), and scar mass was evaluated according to the weight of the sectioned tissue.

In order to evaluate fibrosis, tissue samples were obtained from the infarct area, the border zone and the remote zone, fixed in 10% formalin, sectioned (~8μm thick) and stained with Masson’s trichrome (Sigma Aldrich, HT1079) (n = 5 animals in each group). Five to ten different images (25X) from each region were visualized and captured under light microscopy. The percentage of fibrotic tissue per image was analyzed using ImageJ software. The border zone was defined as the area of normal myocardium at the edge of the scar.

To measure vessel density, sections were stained for Isolectin-B4 (Life Technologies I214111) and smooth muscle actin (sma, Abcam ab5694). Images from the infarct zone, the border zone and the remote zone were captured (Leica Microsystems) and analyzed with Las AF Lite software. Approximately 5–10 images (20X) per region per animal were included in the analysis.

Cardiomyocyte diameter was measured in the border and the remote zones in sections stained with wheat germ agglutinin (to define cell borders; WGA, Invitrogen) and a-sarcomeric actinin (a-sa, Abcam). Five to ten high power field images per section from n = 5 animals per group were imaged (Leica Microsystems) and analyzed. Cells with clearly central nuclei in cross-section, surrounded by sarcomeres were considered cardiomyocytes and were included in the analysis.

To evaluate immune rejection after cell infusion, hematoxylin & eosin-stained sections were analyzed by a pathologist blinded to study group allocation (DL). The infarct zone, the border zone and the remote zone were separately analyzed and graded as to the extent of mononuclear infiltration (none, mild, moderate or severe; 0, 1, 2 and 3 respectively).

### Statistical Analysis

Data were analyzed using GraphPad Prism software (version 5.00, GraphPad, San Diego, California). Data are presented as mean and SEM unless otherwise reported. Changes from baseline to 4 weeks were analyzed with paired samples *t* test. Intergroup comparisons were made using Kruskal Wallis followed by Dunn’s multiple comparison testp ≤ 0.05 was considered statistically significant.

## Results

### Adverse Events and Mortality

Of the total of 42 animals studied, 4 died within 24 hrs of MI induction and 1 was excluded due to post-MI EF>50%. Three animals were used for peripheral blood mononuclear cells (PBMC) injections and three animals to evaluate three-vessel CDC engraftment. One animal infused with CDCs under continuous-flow in all 3 vessels was excluded from paired MRI analysis due to triggering artifacts in the baseline study. The experimental design is depicted in S1 Fig.

### Single Vessel Study

No animals infused with CDCs or placebo died; all completed the protocol of continuous or stop-flow infusion (Fig 1A and 1B). No arrhythmias were observed during single-vessel infusions of either CDCs (n = 10) or placebo (n = 5), with TIMI grade flow = 3 after all infusions. Cardiac TnI levels 24hrs post-infusion remained close to the baseline (<0.05 ng/ml) (Fig 1C and 1D) in the continuous-flow CDC group; in the placebo group, one animal had a significant increase in TnI (3.05ng/ml); and in the stop-flow CDC group, TnI levels increased in 4 of the 5 animals infused (p = 0.56, ANOVA, among all three groups).

#### Functional Analysis

**Fig 2:**
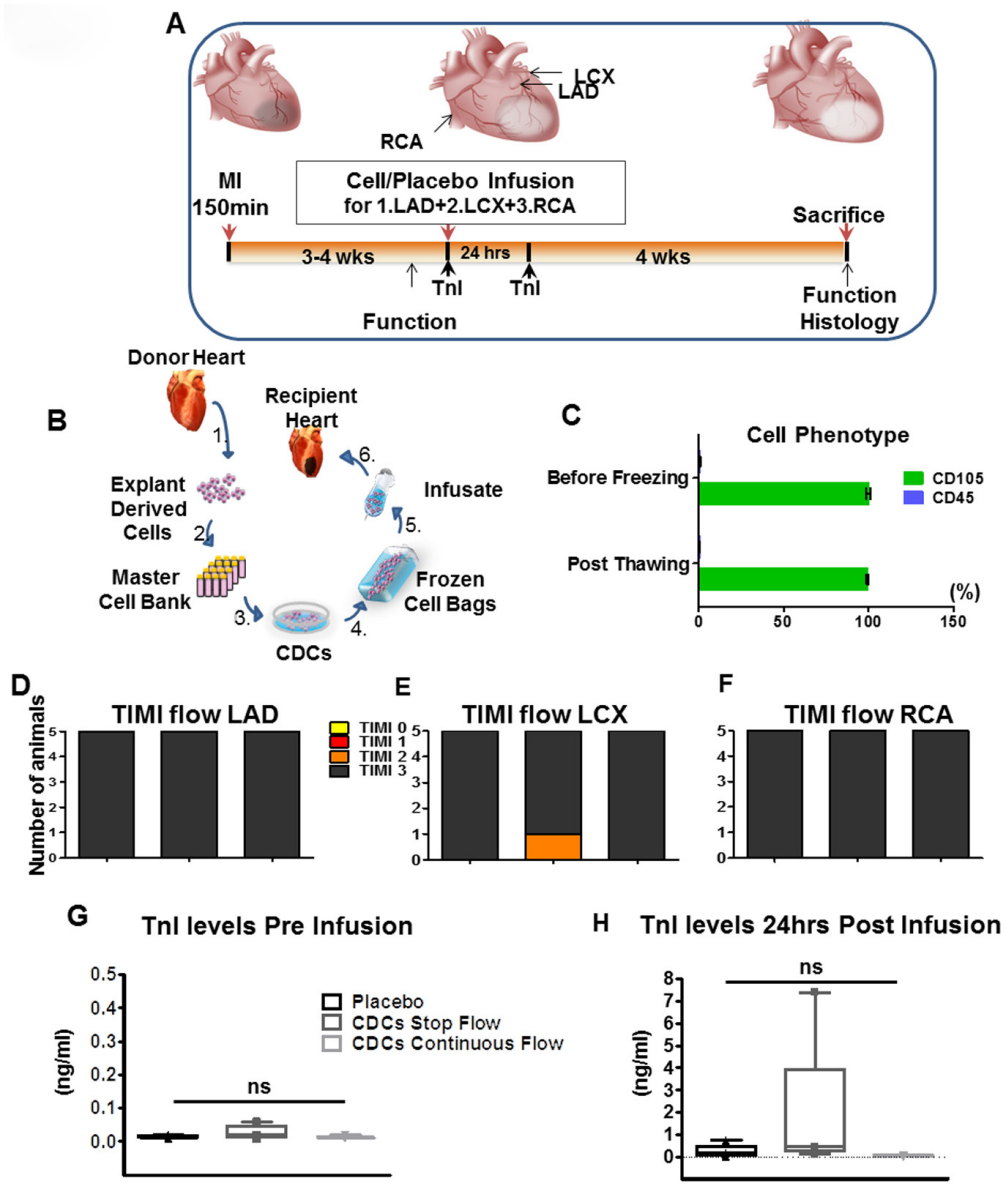
Multi-vessel study design and safety. **A**. Timeline of the study. **B**., Allogeneic CDC isolation and manufacturing from 2 male donor hearts resulted in a master cell bank and bags of frozen CDCs. **C**. Effects of cryopreservation on CD105 and CD45 expression of CDCs. **D., E., F**. TIMI flow post-infusion in each vessel. **G., H**. TnI bump (7.4ng/ml) was observed at 24hrs in one animal after the stop-flow protocol. Error bars indicate SEM. Abbreviations: MI, myocardial infarction; CDCs, cardiosphere-derived cells; MRI, magnetic resonance imaging; LAD: left anterior descending artery.

MRI parameters were equivalent at baseline among groups (Table 1; p = ns for all the baseline parameters by Kruskal Wallis); however, at one month, LV ejection fraction decreased in the placebo compared to either CDC-treated group (ΔEF = -6.9+/-1% in the placebo (median = -5.7; IQR = 4.1), vs -2.76+/-1.1% in the CDC stop-flow (median = -2.1; IQR = 4.75), vs -1.88+/-0.8% in the CDC continuous-flow group (median = -1.3; IQR = 3.5), p = 0.02, Kruskal Wallis; Fig 1E and 1F) due primarily to changes in end-systolic volume (ΔLVESV = +16.18+/-3ml in the placebo (median = 15.6; IQR = 19.5), vs +5.2+/-2.2ml in the CDC stop-flow (median = 5.4; IQR = 8.95), vs +7.7+/-0.6ml in the CDC continuous-flow group (median = 7.2; IQR = 3.2), p = 0.024, Kruskal Wallis; Fig 1G). A similar trend was apparent for LVEDV (ΔLVEDV = +16.4+/-4 ml in the placebo (median = 13; IQR = 17.8), vs +5.26+/-2.9 ml in the CDC stop-flow (median = 4; IQR = 12.25), vs +10.46+/-1.7 ml in the CDC continuous-flow group (median = 11; IQR = 8), p = 0.1, Kruskal Wallis; Fig 1H). Scar mass decreased in CDC-treated animals compared to placebo (Δscar mass = 0.82+/-0.45gr in the placebo (median = 1.1; IQR = 1.9), vs -0.66+/-0.58gr in the CDC stop-flow group (median = -1; IQR = 2.3), vs -1.68+/-0.25gr in the CDC continuous-flow (median = -1.7; IQR = 0.95), p = 0.02, Kruskal Wallis; Fig 1I), but no differences were evident in viable mass at this time point. Regional infarct wall function improved in both treated groups compared to placebo (p = 0.06, Kruskal Wallis; Fig 2H). Infarct wall thickness did not differ in the three groups (p = ns, Kruskal Wallis; Fig 1K).

**Table 1:** Detailed list of MRI-measured parameters for each experimental animal in the Single vessel study (EF: ejection fraction; EDV: End-diastolic volume; ESV: End-systolic volume.

	Baseline						Final					
	EF	EDV	ESV	Scar size	Viable mass	Scar mass	EF	EDV	ESV	Scar size	Scar mass	Viable mass
**LAD continuous flow CDCs**												
**12P177**	43.2	76.8	43.6	17	61.3	10.7	41.9	91.8	53.3	12	8.4	59.3
**12P183**	46.6	72.4	38.6	13	49.3	8.7	42.3	79.3	45.8	10.4	6.7	56.5
**12P184**	36.4	79.1	50.3	19	49.4	13.6	35.3	90.1	58.7	13.3	11.9	58.5
**12P189**	43.4	77.4	43.8	17	52.2	10.3	43.9	90.7	50.9	12.2	9.5	66.7
**13P12**	48	78.3	40.7	14	56	12.3	44.8	84.4	46.6	12.8	10.7	53.6
**LAD stop-flow CDCs**												
**11P183**	50.1	76.2	38	13.45	53.8	8.7	49.5	79	39.9	11.2	7.6	59.1
**11P185**	45.5	84.6	46.1	16.2	57.7	10.7	39.2	96	58.4	15.9	11	58.7
**11P186**	45.2	74.3	40.7	15.97	56	9.1	44.7	86.3	47.7	16	10	65.3
**11P188**	46	80	43.2	22.52	58.4	11.4	43.9	76.1	42.7	16.3	9	58.6
**11P198**	51.6	78.9	38.2	17.4	50	10.6	47.3	82.9	43.6	14.1	9.6	58.3
**LAD placebo**												
**13P48**	40.5	86.3	51.4	18.8	45.17	9.9	35.1	112.5	73	17.6	9.7	57.4
**13P49**	49.2	74.5	37.8	16.1	56.1	9.1	43.5	87.5	49.4	16.1	10.2	49.5
**13P50**	47.7	92.2	48.2	13.1	59.2	10.2	38	102.9	63.8	11	10	65
**13P43**	40.3	67.9	40.5	19.3	47.11	12.2	31	94	65	19.7	13.5	54
**13P44**	46.3	93.1	50	17.6	55.02	10.1	41.8	99	57.6	15	12.2	59

### Multi Vessel Study

#### Phenotypic and viability analysis of CDCs

The study design is depicted in Fig 2A. Based on previous dose-finding data, [11] we chose a per-vessel infusate dose of 12.5 Million CDCs. Cells were manufactured as described above and as depicted in Fig 2B. Uniform expression of CD105 (99.9±1.27%) and vanishingly low expression of CD45 (0.59±0.34%) were detected before freezing and after thawing (Fig 2C). Cell viability also remained high (94±3.2% in the infusate).

#### Study Safety

No animals died during infusion of either CDCs or placebo. In the event of arrhythmias during stop-flow infusion of CDCs or placebo, the procedure in that vessel (LCX: n = 2; RCA: n = 4; Table 2) was terminated and we proceeded to the next vessel, if applicable. A total of 32.35+/- 6.5 Million CDCs ended up being infused in the CDC group (Table 3). No arrhythmias or any other complication were observed during continuous-flow CDC infusions in any of the vessels (Table 2), enabling infusion of a total of 37.1+/-1.2 Million CDCs (Table 3). Compromised TIMI 2 flow was observed in a single animal after LCX CDC infusion under stop-flow conditions (Fig 2D–2F). TnI levels within the expected peri-procedural range [15] were seen in all but one animal in the stop-flow CDC group (TnI = 7.4ng/ml) (Fig 2G and 2H).

**Table 2:** Adverse events during the creation of the MI, during the infusion of either the CDCs or the placebo and post infusions in the multi-vessel study.

Animal #	During MI	LAD INFUSION	LCX INFUSION	RCA INFUSION	POST INFUSION
***Placebo Stop Flow***					
12P62	NONE	NONE	NONE	NONE	NONE
12P100	NONE	NONE	NONE	NONE	NONE
12P101	NONE	NONE	NONE	NONE	NONE
12P193	NONE	NONE	NONE	NSVT post balloon deflation	NONE
12P207	NSVT/VT/VF	NONE	NONE	VT	NONE
***CDCs Stop Flow***					
12P97	NSVT	NONE	BP DROP/VF	BP DROP	NONE
13P31	VT/VF	NONE	NONE	VT/VF	NONE
12P190	NSVT	NONE	NONE	BRADYCARDIA	NONE
12P210	NONE	NONE	NONE	BRADYCARDIA	NONE
13P13	NONE	NONE	VT	NONE	NONE
***CDCs Continuous Flow***					
12P214	NSVT	NONE	NONE	NONE	NONE
13P7	NONE	NONE	NONE	NONE	NONE
12P215	NONE	NONE	NONE	NONE	NONE
13P29	NSVT	NONE	NONE	NONE	NONE
13P10	VT	NONE	NONE	NONE	NONE

**Table 3:** Number of total cells infused in each of the coronary vessels in the multi-vessel study.

Number of cells infused (Millions)			
**CDCs Stop-Flow Infusion**	**LAD**	**LCX**	**RCA**
12P97	12.5	8.3	1.3
13P31	13.2	13.1	4.25
12P190	12.6	13.0	13.1
12P210	12.7	11.9	12
13P13	12.7	8.2	12.9
**CDCs Non-Occlusive FLOW Infusion**	**LAD**	**LCX**	**RCA**
12P214	12.2	11.3	12.9
13P7	12.7	12.2	11.7
12P215	11.7	12.1	12.9
13P29	12.8	13	13.5
13P10	12.1	12.1	12.3

#### MRI measurements at 4 weeks of follow up

**Fig 3:**
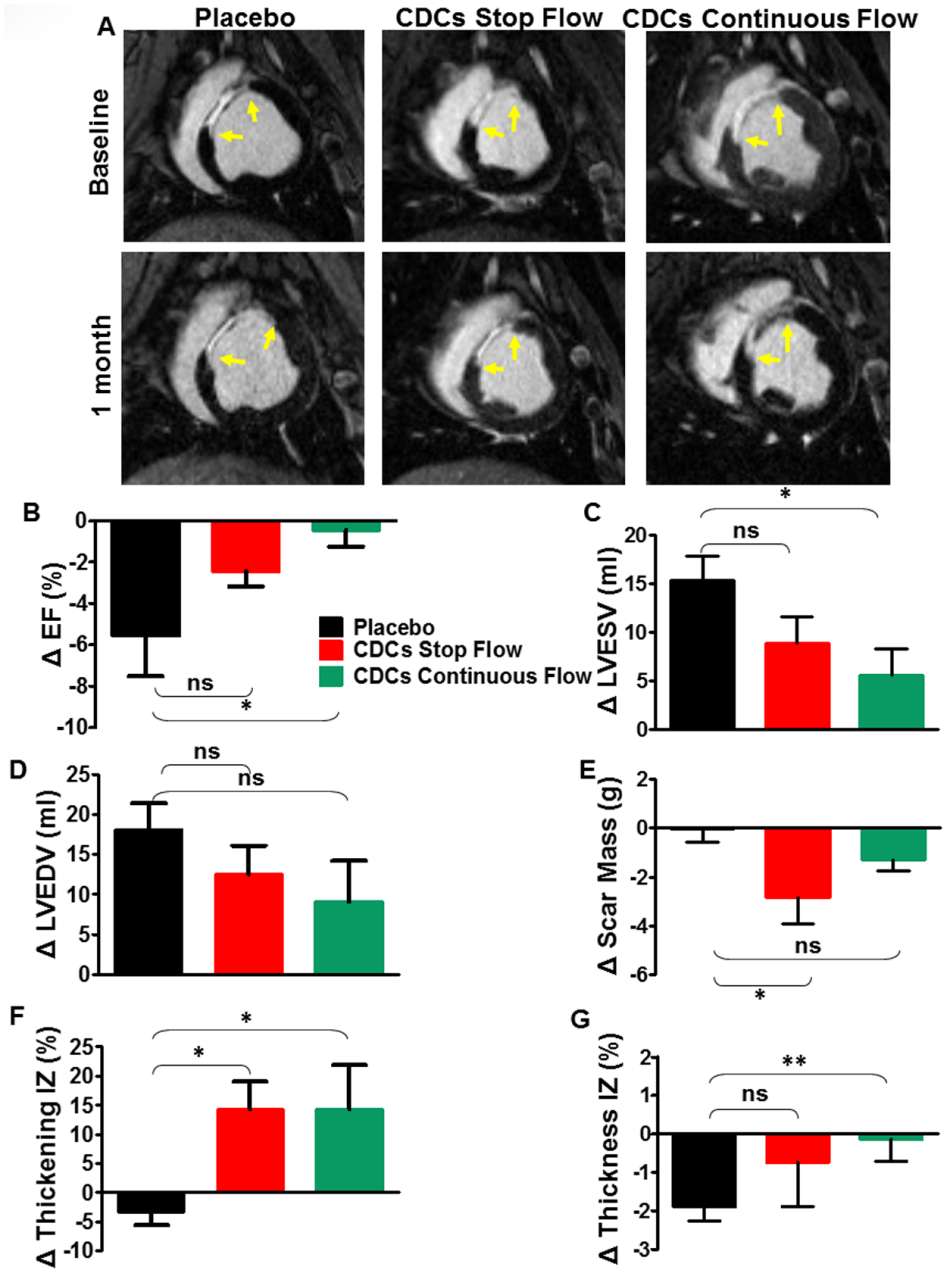
MRI results in triple-vessel protocol. **A**. Representative baseline (upper row) and 1 month post-infusion (lower row) short-axis images in all three groups highlighting the decrease in the scar size and the scar thickness. Yellow arrowheads indicate the infarct zone borders. **B**. Ejection fraction (EF) decreased significantly in the placebo group compared to both CDC-treated groups (p = 0.1 placebo vs CDCs stop-flow and p = 0.05 placebo vs CDCs continuous-flow). **C., D., E**. Changes in end-systolic and end-diastolic volumes and in scar mass in the treated groups compared to placebo. **F**. Infarct wall thickening (p = 0.01 placebo vs CDCs stop-flow and p = 0.05 placebo vs CDCs continuous-flow) and **G**., infarct wall thickness (p = 0.1 placebo vs CDCs stop-flow and p<0.01 placebo vs CDCs continuous-flow) in the placebo compared to both treated groups. Error bars indicate SEM. * p<0.05, ** p<0.01

**Fig 4:**
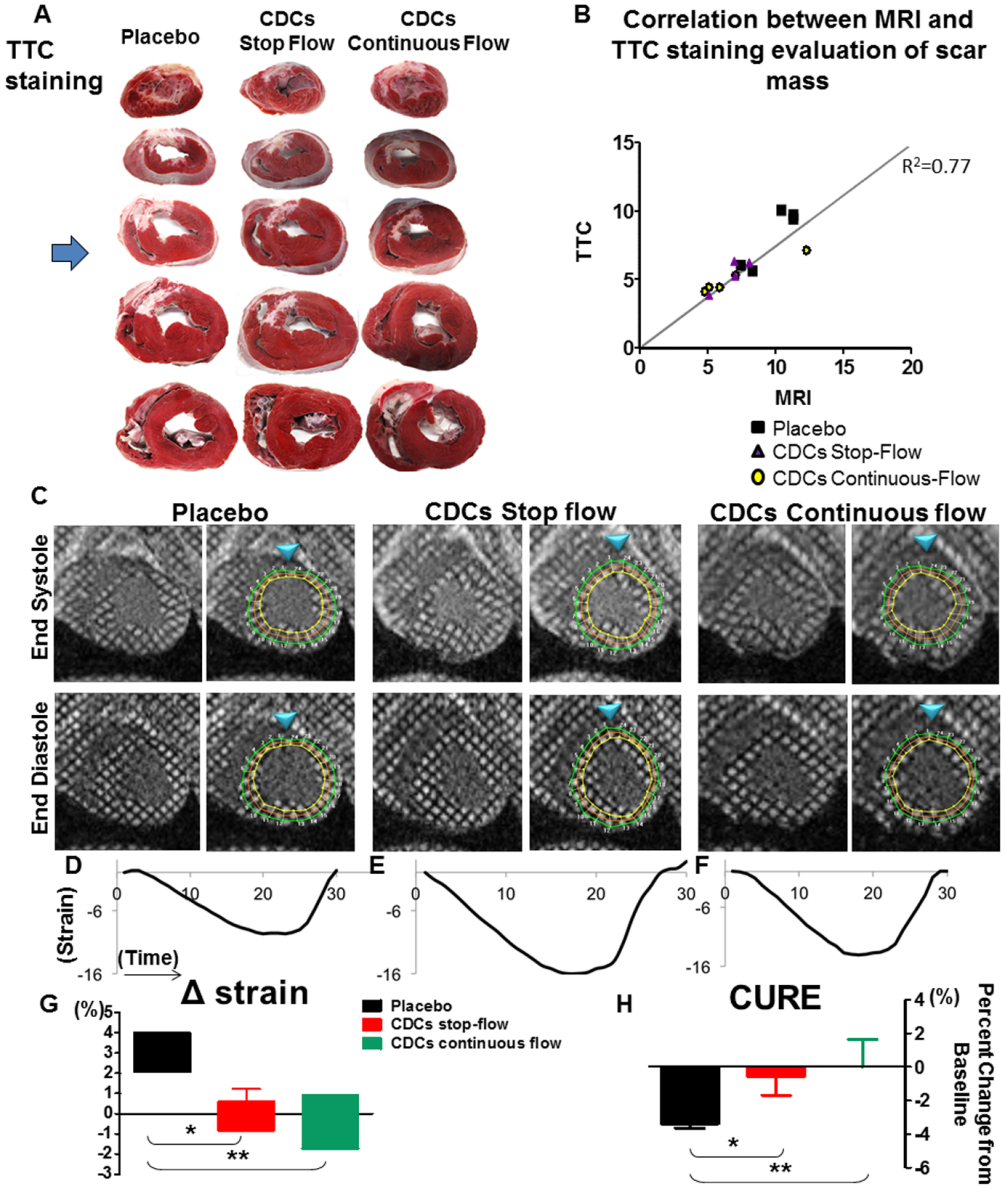
Structure-function correlations. **A**. Representative 2,3,5-triphenyltetrazoliumchloride (TTC) sections covering the LV chamber from the apex to the base from all three groups tested. The blue arrowhead indicates the section level that corresponds to the MR images in Figs 3, 4 and 5. **B**. Correlation between the scar mass (g) evaluated separately by LGE images (JD) and by TTC staining (ET). **C**. Representative tagged CMR images with infarct zone (IZ) defined by the presence of enhancing myocardium, neighboring myocardium as border zone (BZ), and normal myocardium as remote zone (RZ) for the analysis of regional strain, highlighting the contractility differences among the groups. Blue arrowheads indicate the RV insertion and the location of the first segment of each map. **D., E., F**. Representative diagrams of the averaged strain included in the analysis (the more negative, the greater the contractility). **G**. Better contractility was observed in the CDC-treated group compared to the placebo. **H**. Trend towards better effect of CDC therapy on mid-ventricular wall synchrony assessed by CURE ratio. Error bars indicate SEM. * p<0.05, ** p<0.01

MRI parameters were equivalent at baseline among groups (Table 4; p = ns for all baseline parameters by Kruskal Wallis). At one month of follow-up, MRI analysis showed significant preservation of ejection fraction in the CDC-treated animals (ΔEF = -5.8+/-1.8% in the placebo (median = -4.5; IQR = 6.5) vs -2.4+/-0.7% in the CDC stop-flow group (median = -3.2; IQR = 3.1) vs -0.46+/-0.8% in the CDC continuous-flow (median = -0.6; IQR = 2.75), p = 0.038, Kruskal Wallis test; representative MRI images in Fig 3A and pooled data in Fig 3B) due predominantly to changes in end-systolic volume (Δ LVESV +15±2.57% in the placebo (median = 16.3; IQR = 10.85) vs +8.86±2.8% in the stop-flow group (median = 6.2; IQR = 7.75) vs +5.5±2.8% in the continuous-flow group (median = 5.7; IQR = 11.9); p = ns, Kruskal Wallis, but p = 0.41 between placebo and continuous-flow; Fig 3C). A trend towards less expansion was observed in the end-diastolic volumes (Fig 3D). Scar mass reduction was greater in the CDC-treated groups compared to placebo (Δscar mass = -0.04+/-0.5gr in the placebo (median = -0.1; IQR = 2.1) vs -2.8+/-1gr in the CDC stop-flow group (median = -2.6; IQR = 4.4) vs -1.3+/-0.4gr in the CDC continuous-flow (median = -1.7; IQR = 1.89), p = 0.04, Kruskal Wallis; Fig 3E). TTC-stained sections from the same hearts validated the MRI scar mass results (Fig 4A and 4B), further documenting that MRI reliably reports the structural changes that underlie CDC therapeutic benefit.[13] Regional infarct wall motion analysis showed increased wall thickening and decreased wall atrophy in both CDC-treated groups compared to placebo (Fig 3F and 3G). Regional strain by HARP (p = 0.01, Kruskal Wallis; Fig 4C–4G) and mechanical synchrony by CURE (p = 0.1, Kruskal Wallis; Fig 4H) were enhanced in the CDC-treated groups, compared to the placebo but only the former parameter reached significant levels.

**Table 4:** Detailed list of MRI-measured parameters for each experimental animal in the three vessel study (EF: ejection fraction; EDV: End-diastolic volume; ESV: End-systolic volume.

	Baseline						Final					
	EF	EDV	ESV	Scar size	Viable mass	Scar mass	EF	EDV	ESV	Scar size	Scar mass	Viable mass
**Placebo**												
12P62	49.1	65.8	33.7	20	71.59	10	36.5	88.6	56.2	16	11.4	60.2
12P100	49.4	65.7	33.2	20	68.87	11.7	43.8	73.1	44.5	12	9.8	62.5
12P101	41	67.7	39.9	19	65.03	10.4	36.5	88.5	56.2	16.4	11	56.1
12P193	45.3	70	38	15	65.9	7.9	40.8	95.4	56.5	13.8	7.7	65.3
12P207	46.1	65.8	36.6	14	55.76	6.7	44	80	44.6	12.7	6.6	51.6
**3 vessel stop flow**												
**12P97**	46.9	79	42	16	70.66	15.1	46.6	91.4	48.8	11	8.6	69.3
**12P190**	47.2	68.2	36	19	64.21	9.8	46.1	78.4	42.2	11.4	7.2	63.2
**12P210**	46.2	76.1	40.9	14	61.09	8.6	42.2	80.9	46.7	12.2	7.8	50.7
**13P13**	60.1	66.6	27.1	9	70.25	6	56.9	75.6	32.6	6.7	5.4	72.2
**13P31**	39.9	91.8	55.2	21	70	17.7	36.3	117.9	75.2	15.9	14	58.8
**3 vessel continuous flow**												
**12P214**	50.7	53.4	26.3	16	55.8	9.2	53.1	68.3	32	11.4	7.11	55.5
**13P7**	51.5	69	33.5	11	60.54	6.4	51	72.4	35.5	8.2	4.2	64
**12P215**	40.9	94.6	55.9	17	59.46	10.7	40.3	112.8	67.4	16	10.3	67.3
**13P10**	47.8	77.3	40.4	14	65.31	7.4	46.4	69	37.5	11	7.3	69.9
**13P29**	45.9	74.3	40.2	15	60.63	10.2	43.7	91.6	51.6	13	8.5	59.8

#### Regional function comparison between the single-vessel and the three-vessel study

**Fig 5:**
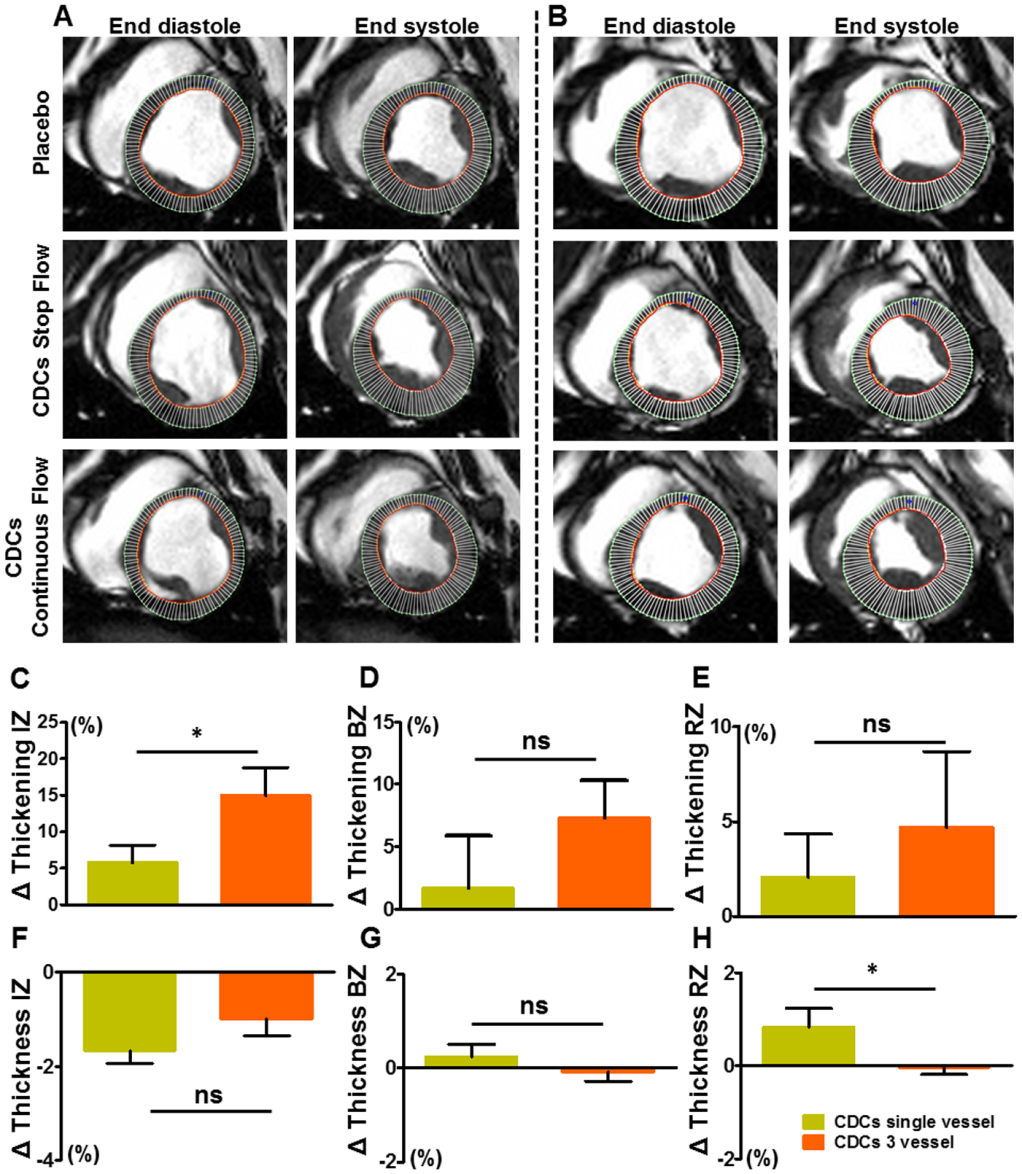
Regional wall motion comparison. **A**. Representative short axis images at 1 month of follow up in end-systole and end-diastole in the single-vessel study and **B**., in the multi-vessel study. A mesh drawn according to defined epicardial and endocardial contours divides each tissue section into 100 segments. **C.,-H**. Paired analysis of regional thickening and thickness at 1 month of follow up in single-vessel and 3-vessel CDC groups. Error bars indicate SEM. Abbreviations: IZ, infarct zone; BZ, border zone; RZ, remote zone. * p<0.05.

Serial intergroup analysis between the single-vessel (n = 10) and three-vessel (n = 10) CDC-treated groups revealed a trend for superiority of multi-vessel groups in all parameters for regional function, which reached significance for infarct zone thickening (p = 0.04) and remote zone thickness (p = 0.03, Fig 5).

#### Histopathological evaluation and tissue architecture

**Fig 6:**
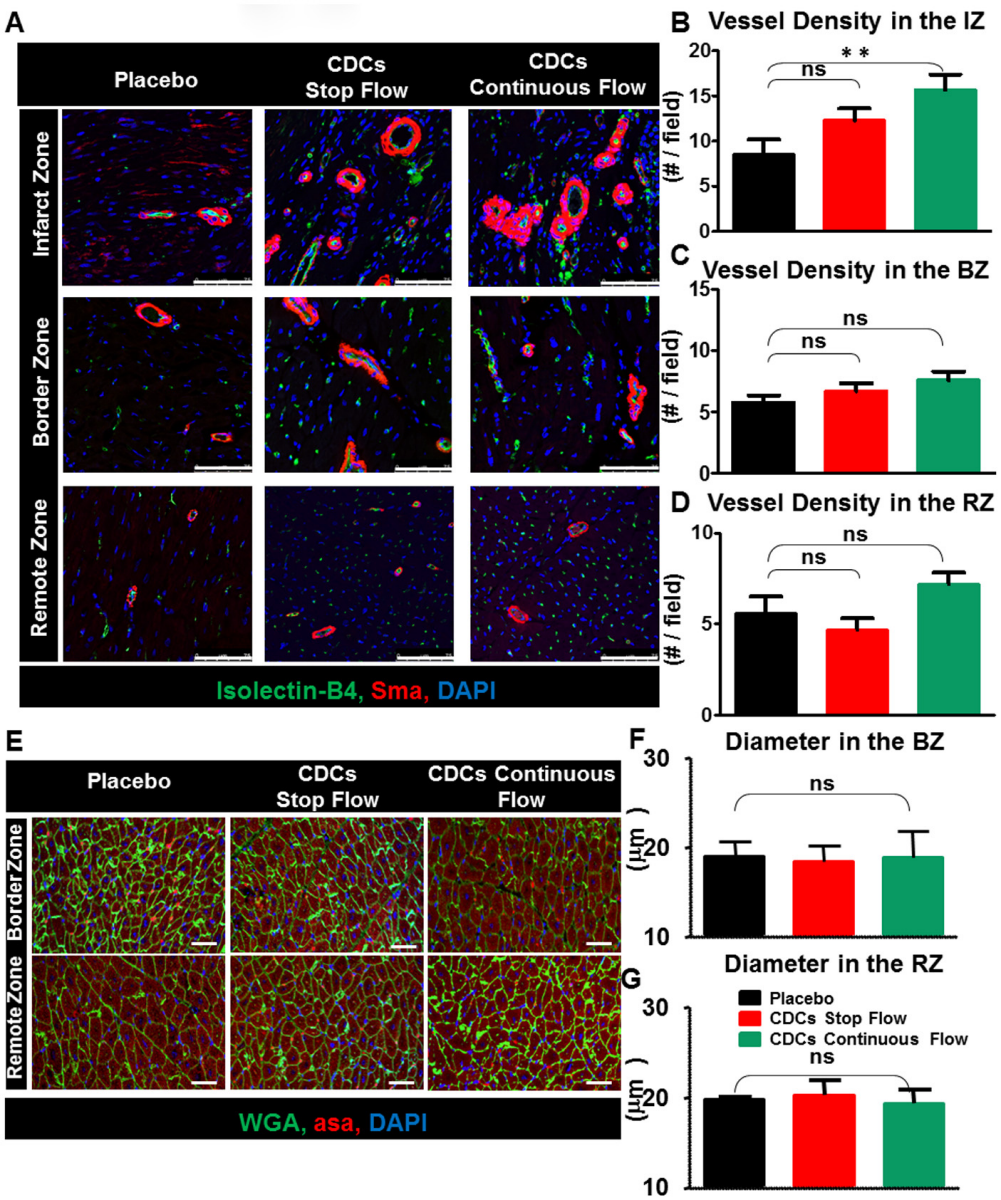
Vessel density and cardiomyocyte hypertrophy. **A**. Representative immunostaining sections with sma, isolectin B4 and DAPI, 1 month post treatment. **B**. Vessel density within the IZ (p = 0.05), **C**., the BZ and **D**., the RZ in the CDC-treated compared to placebo (Scale bar = 75μm). **E**. Representative sections immunostained with α-sa and WGA. **F., G**. Similar cardiomyocyte diameters in all three groups, both in the BZ and in the RZ. Scale bar = 50μm. Abbreviations: IZ, infarct zone; BZ, border zone; RZ, remote zone; sma, smooth muscle actin (red); isolectin B4 (green); DAPI, blue, 4',6-diamidino-2-phenylindole; asa, α-sarcomeric actinin; WGA, wheat germ agglutinin to define the cell borders. Error bars indicate SEM.

Serial tissue staining for regional architectural changes associated with the CDC-induced functional remodeling [21–23] revealed enhanced vascularity (sma+/Isolectin+) only within the infarct zone (Fig 6A–6D). Cardiomyocyte diameter remained similar among all groups analyzed (Fig 6E–6G).

Significantly less fibrosis was detected within the infarct zone and the border zone of the CDC-treated groups compared to the placebo, with a similar trend in the remote myocardium (S2A–S2D Fig). Immunologically, modestly increased lymphocytic infiltration was reported only in the infarct zone post CDCs compared to placebo. The border and remote zones showed no or mild lymphocytic infiltration (S2E–S2H Fig).

Taken together, the histology data show that triple doses of allogeneic CDCs boosted neovascularization and reduced regional fibrosis at 1 month of follow-up, without detrimental inflammatory consequences.

#### Short Term Engraftment

Luciferase-expressing CDCs (12.5M) infused in each of the three coronary vessels under continuous-flow (n = 3) revealed activity in the infarct zone at a level similar to that previously reported under single-vessel stop-flow [11,13]; unlike single-vessel delivery, however, three-vessel infusion also led to sizable engraftment in the border and remote zones, [11] areas supplied by the LCX and RCA (S3A–S3D Fig). Thus, three-vessel continuous-flow delivery achieves widespread myocardial distribution of infused CDCs.

#### Circulating alloantibodies

Four weeks post cell infusion, serum alloantibodies were detectable only in 1 of the 8 animals evaluated at this time point (S3E and S3F Fig), consistent with previous studies using CDCs in small and large animals without alloantibodies after single doses [13,21]. Further larger studies are needed to validate our findings, and particularly to investigate the possibility of allosensitization with repeat dosing.

## Discussion

### Stop-flow versus continuous-flow

Here we have assessed various delivery methods for cardiac cell therapy, with regard to safety and effects on global LV remodeling post-MI. While the safety and the efficacy of CDC delivery through stop-flow infusion were recently validated in humans in the CADUCEUS study,[12] the need for stop-flow delivery is unclear. In order to address whether balloon inflation is actually needed for efficacious delivery of CDCs, we systematically compared intracoronary cell delivery with and without balloon inflations. Both methods were equally effective in reducing scar and attenuating LV remodeling, but post-procedural TnI leak was much less common with continuous- flow. The results call into question the need for balloon inflation, at least with CDCs (which are fairly large: average diameter ~19 μm [11]) although continuous flow also appears to work with other cell types [24,25]. The documented ability of infused CDCs to cross the vascular wall under non-occlusive conditions [26,27] has been previously demonstrated in small-animal studies and further rationalizes our findings.

### One vessel versus three vessels

To test the effect of CDCs on global LV remodeling, we performed a multi-vessel infusion study with MRI and histology as endpoints. MRI revealed that CDCs reduce scar mass (Fig 3E), a finding which was validated by TTC staining of explanted hearts (Fig 4A and 4B). These changes in tissue structure were accompanied by better-preserved EF in the treated groups compared to placebo, and by improved regional wall motion (Figs 3B and 4G), evaluated separately with tagged MRI analysis which can quantify accurately the deformation of the LV throughout systole and diastole [17,19]. Other studies have focused on quantifying cellular benefit with EF and scar size changes, [28–31] with inconsistent results [32]. Here, the superiority of triple-vessel infusion relative to single-vessel infusion was evident on regional wall motion (Fig 5C–5H) despite the fact that only one vascular territory had been infarcted. Our data in a post-MI heart failure model are in line with previous data in a porcine model of hibernating myocardium [33]. More specifically, Suzuki et al showed that global intracoronary infusion of CDCs can attenuate adverse remodeling. The confirmation of bioactivity in two complementary disease models increases confidence in the potential clinical relevance of this therapeutic strategy.

### Three-vessel intracoronary delivery versus direct intramyocardial injection

**Fig 7:**
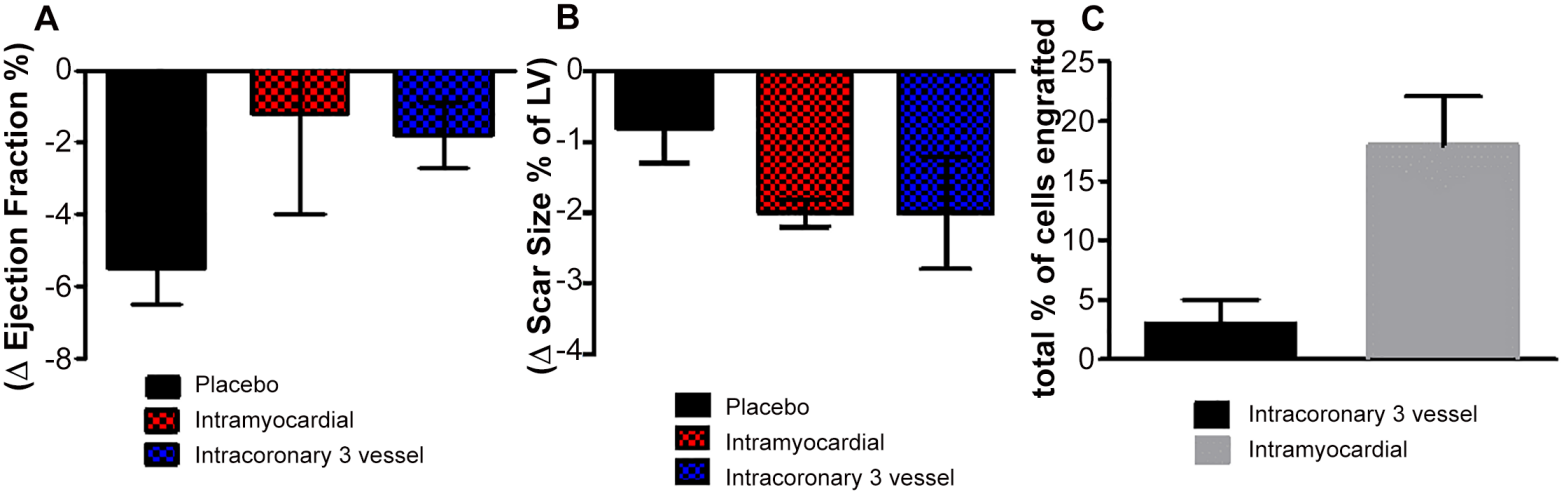
Side-by-side evaluation of intramyocardial injections and intracoronary infusions of CDCs data. **A**. The cell treatment effect of both intramyocardial and intracoronary delivery was superior to placebo with no difference between the 2 treatment groups. **B**. Similar changes were observed in scar size reduction. **C**. More cells were detected post intramyocardial injections compared to intracoronary delivery at 24hr of evaluation. Intracoronary data from the present study; intramyocardial data replotted from ref. 32. Error bars indicate SEM.

Another means of attempting to achieve broad myocardial coverage with cell transplantation involves the use of injection catheters, with cells applied at 10–15 distinct intramyocardial sites [34–36]. Unlike the IC route, intramyocardial injection is not used in clinical practice, and it is technically much more challenging [33, 37–40]. Nevertheless, if outcomes were clearly superior, the risks and difficulties of intramyocardial injection might be defensible [41]. To gauge the relative efficacy of the two approaches, we compared the present results using 3-vessel IC delivery to prior work from our lab using intramyocardial injections of CDCs [35]. Fig 7 shows the treatment-related changes in EF (A), scar size (B) and engraftment (C). While engraftment at 24 hrs was much greater with intramyocardial injection (C),[41,42] functional benefits (A) and reductions in scar size (B) were comparable with the two approaches. Thus, this heuristic comparison reveals no particular advantage of intramyocardial injection relative to IC delivery. But, a dedicated prospective comparison of the two methods would be required to reach definitive conclusions.

### Mechanisms of CDC benefit

The safety and efficacy of allogeneic CDCs post-MI and in hibernating myocardium have been well-established in preclinical models [13,21,33]. CDCs secrete numerous beneficial cytokines, growth factors and exosomes laden with microRNAs which contribute to the observed benefits [23,43,44]. In the long-term efficacy studies performed to date, CDC therapy induces reductions in tissue fibrosis, while promoting angiogenesis and the recruitment of endogenous cardiac progenitors[21,22,45–49]. Here, global CDC infusion boosted global short-term cell engraftment and angiogenesis, with greater reductions in fibrosis in the border zone compared to single-vessel infusion [11]. Gadolinium kinetics are not impaired by the enhanced vasculogenesis, nor does the latter undermine late enhancement image quantification for scar size,[13] increasing confidence in the MRI findings (which are nevertheless confirmed histologically here; Fig 4A and 4B).

Our study has several limitations. First of all, the anterior MI model is not ideally suited for the investigation of cardiomyopathy, as the injury is focal and systemic heart failure is not prominent. The paucity of myocardial changes in remote zones may lead to an underestimate of the therapeutic potential of CDCs in generalized myocardial disorders [49]. The one month post-treatment follow-up is also likely to minimize the detection of therapeutic efficacy, as the effects of CDCs are known to increase over time [12,13]. Another limitation is the lack of a placebo multi-vessel group under continuous flow. Nevertheless, the findings of safety and improved efficacy in this well-studied model give good reason to question the need for stop-flow intracoronary infusion, and highlight the benefits (including broader myocardial coverage) of multi-vessel infusion.

In conclusion, we have addressed a number of issues that are central to the delivery of cell therapy (safety and efficacy of stop-flow versus continuous-flow, and of single- versus triple-vessel infusion). Our findings give reason to believe that global cell infusion may be a promising translational tool, particularly to treat generalized cardiac disorders. These data provide preclinical validation for nonocclusive multi-vessel cell delivery, as is being utilized in the phase 2a DYNAMIC (**D**ilated cardiom**Y**opathy i**N**tervention with **A**llogeneic **M**yocard**I**ally-regenerative **C**ells) trial of allogeneic CDCs in patients with ischemic and non-ischemic heart failure (clinicaltrials.gov).

## Supporting Information

S1 Fig Experimental Design. A total of 42 Yucatan mini-pigs were used in the current study including 2 substudies: n = 15 animals were infused only in the LAD and n = 19 animals were infused in all three coronary vessels.
(TIF)

S2 Fig Histopathological analysis post cell therapy. **A**. Representative Masson’s trichrome stained sections. **B**. The fibrotic tissue was significantly attenuated within the IZ and **C**., the BZ but not **D**., the RZ in both treated groups compared to the placebo. **E**. Representative hematoxylin & eosin stained sections. **F., G., H**. No significant mononuclear infiltration was observed 1 month post cell infusion compared to placebo. Error bars indicate SEM. Abbreviations: IZ, infarct zone; BZ, border zone; RZ, remote zone. Scale bar = 50μm. * p<0.05.
(TIF)

S3 Fig Engraftment of luciferase-transduced CDCs after multi-vessel continuous-flow infusion. **A**. Scheme of the transduction and **B**. The timeline of the engraftment experiment. 12.5M CDCs were infused sequentially in each of the three coronary vessels. **C**. Strong Luc+ expression was found in the LAD and the LCX territories 24hrs post intracoronary infusion. Somewhat fewer cells engrafted in the RCA territory, but the differences among groups are not significant. **D**. Careful segmental analysis revealed that the anterior infarcted wall and the LCX territory received the majority of the Luc+ cells. **E., F**. Minimal detection of circulating alloantibodies post CDC infusion but the experiment is limited due to the lack of antibody quantification pre infusion. On the left plot, the red curve defines the negative control sample, the black curve the positive control and all the rest curves are the pig samples tested. Error bars indicate SEM.
(TIF)

## References

[1] Lara-Pezzi E, Menasche P, Trouvin JH, Badimon L, Ioannidis JP, Wu JC et al (2015) Guidelines for translational research in heart failure. Journal of cardiovascular translational research 8(1):3–22. 10.1007/s12265-015-9606-8 25604959

[2] Stamm C, Nasseri B, Choi YH, Hetzer R. (2009) Cell therapy for heart disease: Great expectations, as yet unmet. Heart, lung & circulation 18(4):245–56. 10.1016/j.hlc.2008.10.014 19119076

[3] Jeevanantham V, Butler M, Saad A, Abdel-Latif A, Zuba-Surma EK, Dawn B. (2012) Adult bone marrow cell therapy improves survival and induces long-term improvement in cardiac parameters: A systematic review and meta-analysis. Circulation 126(5):551–68. 10.1161/CIRCULATIONAHA.111.086074 Epub 2012 Jun 22. Review. 22730444 PMC4282649

[4] Assmus B, Schachinger V, Teupe C, Britten M, Lehmann R, Dobert N et al (2002) Transplantation of progenitor cells and regeneration enhancement in acute myocardial infarction (topcare-ami). Circulation 106:3009–3017. 12473544 10.1161/01.cir.0000043246.74879.cd

[5] Behfar A, Crespo-Diaz R, Terzic A, Gersh BJ. (2014) Cell therapy for cardiac repair—lessons from clinical trials. Nature reviews. Cardiology 11(4):232–46. 10.1038/nrcardio.2014.9 Review. 24594893

[6] Rosen MR, Myerburg RJ, Francis DP, Cole GD, Marban E. (2014) Translating stem cell research to cardiac disease therapies: Pitfalls and prospects for improvement. J Am Coll Cardiol 64(9):922–37. 10.1016/j.jacc.2014.06.1175 25169179 PMC4209166

[7] Malliaras K, Kreke M, Marban E. (2011) The stuttering progress of cell therapy for heart disease. Clin Pharmacol Ther 90(4):532–41. 10.1038/clpt.2011.175 21900888

[8] Rasmussen TL, Raveendran G, Zhang J, Garry DJ. (2011) Getting to the heart of myocardial stem cells and cell therapy. Circulation 123(16):1771–9. 10.1161/CIRCULATIONAHA.109.858019 21518990 PMC3547391

[9] Ye L, Chang YH, Xiong Q, Zhang P, Zhang L, Somasundaram P et al (2014) Cardiac repair in a porcine model of acute myocardial infarction with human induced pluripotent stem cell-derived cardiovascular cells. Cell stem cell 15(6):750–61. 10.1016/j.stem.2014.11.009 25479750 PMC4275050

[10] Smith RR, Barile L, Cho HC, Leppo MK, Hare JM, Messina E et al (2007) Regenerative potential of cardiosphere-derived cells expanded from percutaneous endomyocardial biopsy specimens. Circulation 115:896–908. 10.1161/CIRCULATIONAHA.106.655209 17283259

[11] Johnston PV, Sasano T, Mills K, Evers R, Lee ST, Smith RR et al (2007) Engraftment, differentiation, and functional benefits of autologous cardiosphere-derived cells in porcine ischemic cardiomyopathy. Circulation 120(12):1075–83, 7 p following 1083. 10.1161/CIRCULATIONAHA.108.816058 PMC2848167 19738142

[12] Makkar RR, Smith RR, Cheng K, Malliaras K, Thomson LE, Berman D et al (2012) Intracoronary cardiosphere-derived cells for heart regeneration after myocardial infarction (caduceus): A prospective, randomised phase 1 trial. Lancet 379(9819):895–904. 10.1016/S0140-6736(12)60195-0 22336189 PMC4326004

[13] Malliaras K, Smith RR, Kanazawa H, Yee K, Seinfeld J, Tseliou E et al (2013) Validation of contrast-enhanced magnetic resonance imaging to monitor regenerative efficacy after cell therapy in a porcine model of convalescent myocardial infarction. Circulation 128(25):2764–75. 10.1161/CIRCULATIONAHA.113.002863 24061088 PMC3907064

[14] The thrombolysis in myocardial infarction (timi) trial. Phase i findings. Timi study group. (1985) N Engl J Med 312:932–936 4038784 10.1056/NEJM198504043121437

[15] Povsic TJ, Losordo DW, Story K, Junge CE, Schatz RA, Harrington RA et al (2012) Incidence and clinical significance of cardiac biomarker elevation during stem cell mobilization, apheresis, and intramyocardial delivery: An analysis from act34-cmi. American heart journal1 64(5):689–697.e3. 10.1016/j.ahj.2012.06.022 23137499

[16] Amado LC, Gerber BL, Gupta SN, Rettmann DW, Szarf G, Schock R et al (2004) Accurate and objective infarct sizing by contrast-enhanced magnetic resonance imaging in a canine myocardial infarction model. J Am Coll Cardiol 44:2383–2389. 10.1016/j.jacc.2004.09.020 15607402

[17] Kim RJ, Wu E, Rafael A, Chen EL, Parker MA, Simonetti O et al (2000) The use of contrast-enhanced magnetic resonance imaging to identify reversible myocardial dysfunction. N Engl J Med 343:1445–1453. 10.1056/NEJM200011163432003 11078769

[18] Kali A, Cokic I, Tang RL, Yang HJ, Sharif B, Marban E et al (2014) Determination of location, size, and transmurality of chronic myocardial infarction without exogenous contrast media by using cardiac magnetic resonance imaging at 3 t. Circulation. Cardiovascular imaging 7:471–481. 10.1161/CIRCIMAGING.113.001541 24682268 PMC4077784

[19] Castillo E, Osman NF, Rosen BD, El-Shehaby I, Pan L, Jerosch-Herold M et al (2005) Quantitative assessment of regional myocardial function with mr-tagging in a multi-center study: Interobserver and intraobserver agreement of fast strain analysis with harmonic phase (harp) mri. Journal of cardiovascular magnetic resonance: official journal of the Society for Cardiovascular Magnetic Resonance 7:783–791 16358393 10.1080/10976640500295417

[20] Byrne MJ, Helm RH, Daya S, Osman NF, Halperin HR, Berger RD. (2007) Diminished left ventricular dyssynchrony and impact of resynchronization in failing hearts with right versus left bundle branch block. J Am Coll Cardiol 50:1484–1490. 10.1016/j.jacc.2007.07.011 17919569

[21] Malliaras K, Li TS, Luthringer D, Terrovitis J, Cheng K, Chakravarty T. (2012) Safety and efficacy of allogeneic cell therapy in infarcted rats transplanted with mismatched cardiosphere-derived cells. Circulation 125:100–112. 10.1161/CIRCULATIONAHA.111.042598 22086878 PMC3256094

[22] Tseliou E, Pollan S, Malliaras K, Terrovitis J, Sun B, Galang G et al (2013) Allogeneic cardiospheres safely boost cardiac function and attenuate adverse remodeling after myocardial infarction in immunologically mismatched rat strains. Journal of the American College of Cardiology 61:1108–1119. 10.1016/j.jacc.2012.10.052 23352785

[23] Chimenti I, Smith RR, Li TS, Gerstenblith G, Messina E, Giacomello A et al (2010) Relative roles of direct regeneration versus paracrine effects of human cardiosphere-derived cells transplanted into infarcted mice. Circ Res 106:971–980. 10.1161/CIRCRESAHA.109.210682 20110532 PMC4317351

[24] Vrtovec B, Poglajen G, Lezaic L, Sever M, Domanovic D, Cernelc P et al (2013) Effects of intracoronary cd34+ stem cell transplantation in nonischemic dilated cardiomyopathy patients: 5-year follow-up. Circ Res 112:165–173. 10.1161/CIRCRESAHA.112.276519 23065358

[25] Houtgraaf JH, de Jong R, Kazemi K, de Groot D, van der Spoel TI, Arslan F et al (2013) Intracoronary infusion of allogeneic mesenchymal precursor cells directly after experimental acute myocardial infarction reduces infarct size, abrogates adverse remodeling, and improves cardiac function. Circ Res 113:153–166. 10.1161/CIRCRESAHA.112.300730 23658436

[26] Lee ST, White AJ, Matsushita S, Malliaras K, Steenbergen C, Zhang Y et al (2011) Intramyocardial injection of autologous cardiospheres or cardiosphere-derived cells preserves function and minimizes adverse ventricular remodeling in pigs with heart failure post-myocardial infarction. JACC 57:455–465. 10.1016/j.jacc.2010.07.049 21251587

[27] Cheng K, Shen D, Xie Y, Cingolani E, Malliaras K, Marban E. (2012) Brief report: Mechanism of extravasation of infused stem cells. Stem cells. 30:2835–2842. 10.1002/stem.1184 23135922 PMC3508398

[28] Malliaras K, Marban E. (2014) Moving beyond surrogate endpoints in cell therapy trials for heart disease. Stem Cells Transl Med 3:2–6. 10.5966/sctm.2013-0104 24292794 PMC3902289

[29] Wollert KC, Meyer GP, Lotz J, Ringes-Lichtenberg S, Lippolt P, Breidenbach C et al (2004) Intracoronary autologous bone-marrow cell transfer after myocardial infarction: The boost randomised controlled clinical trial. Lancet. 364:141–148. 10.1016/S0140-6736(04)16626-9 15246726

[30] Meyer GP, Wollert KC, Lotz J, Steffens J, Lippolt P, Fichtner S et al (2006) Intracoronary bone marrow cell transfer after myocardial infarction: Eighteen months' follow-up data from the randomized, controlled boost (bone marrow transfer to enhance st-elevation infarct regeneration) trial. Circulation 113:1287–1294. 10.1161/CIRCULATIONAHA.105.575118 16520413

[31] Herbots L, D'Hooge J, Eroglu E, Thijs D, Ganame J, Claus P et al Improved regional function after autologous bone marrow-derived stem cell transfer in patients with acute myocardial infarction: A randomized, double-blind strain rate imaging study. Eur Heart J. 30:662–670. 10.1093/eurheartj/ehn532 19106196

[32] Traverse JH, Henry TD, Moye LA. (2011) Is the measurement of left ventricular ejection fraction the proper end point for cell therapy trials? An analysis of the effect of bone marrow mononuclear stem cell administration on left ventricular ejection fraction after st-segment elevation myocardial infarction when evaluated by cardiac magnetic resonance imaging. American heart journal 162:671–677. 10.1016/j.ahj.2011.06.019 21982659

[33] Suzuki G, Weil BR, Leiker MM, Ribbeck AE, Young RF, Cimato TR et al (2014) Global intracoronary infusion of allogeneic cardiosphere-derived cells improves ventricular function and stimulates endogenous myocyte regeneration throughout the heart in swine with hibernating myocardium. PLoS One 9:e113009 10.1371/journal.pone.0113009 eCollection 2014. 25402428 PMC4234497

[34] Hare JM, Fishman JE, Gerstenblith G, DiFede Velazquez DL, Zambrano JP, Suncion VY et al (2012) Comparison of allogeneic vs autologous bone marrow-derived mesenchymal stem cells delivered by transendocardial injection in patients with ischemic cardiomyopathy: The poseidon randomized trial. JAMA 308:2369–2379. 10.1001/jama.2012.25321 23117550 PMC4762261

[35] Yee K, Malliaras K, Kanazawa H, Tseliou E, Cheng K, Luthringer DJ et al (2014) Allogeneic cardiospheres delivered via percutaneous transendocardial injection increase viable myocardium, decrease scar size, and attenuate cardiac dilatation in porcine ischemic cardiomyopathy. PLoS One 9:e113805 10.1371/journal.pone.0113805 25460005 PMC4251970

[36] Perin EC, Dohmann HF, Borojevic R, Silva SA, Sousa AL, Mesquita CT et al (2003) Transendocardial, autologous bone marrow cell transplantation for severe, chronic ischemic heart failure. Circulation 107:2294–2302. 10.1161/01.CIR.0000070596.30552.8B 12707230

[37] Fukushima S, Varela-Carver A, Coppen SR, Yamahara K, Felkin LE, Lee J et al (2007) Direct intramyocardial but not intracoronary injection of bone marrow cells induces ventricular arrhythmias in a rat chronic ischemic heart failure model. Circulation 115:2254–2261. 10.1161/CIRCULATIONAHA.106.662577 17438152

[38] Dib N, Khawaja H, Varner S, McCarthy M, Campbell A. (2011) Cell therapy for cardiovascular disease: A comparison of methods of delivery. Journal of cardiovascular translational research. 4:177–181. 10.1007/s12265-010-9253-z 21181320 PMC3047684

[39] Baldazzi F, Jorgensen E, Ripa RS, Kastrup J. (2008) Release of biomarkers of myocardial damage after direct intramyocardial injection of genes and stem cells via the percutaneous transluminal route. Eur Heart J 29:1819–1826. 10.1093/eurheartj/ehn233 18524811

[40] Ye L, Chang YH, Xiong Q, Zhang L, Somasundaram P, Lepley M et al (2014) Cardiac repair in a porcine model of acute myocardial infarction with human induced pluripotent stem cell-derived cardiovascular cells. Cell Stem Cell.15(6):750–61. 10.1016/j.stem.2014.11.009 25479750 PMC4275050

[41] Hou D, Youssef EA, Brinton TJ, Zhang P, Rogers P, Price ET et al (2005) Radiolabeled cell distribution after intramyocardial, intracoronary, and interstitial retrograde coronary venous delivery: Implications for current clinical trials. Circulation 112:I150–156. 16159808 10.1161/CIRCULATIONAHA.104.526749

[42] Perin EC, Silva GV, Assad JA, Vela D, Buja LM, Sousa AL et al (2008) Comparison of intracoronary and transendocardial delivery of allogeneic mesenchymal cells in a canine model of acute myocardial infarction. Journal of molecular and cellular cardiology 44:486–495. 10.1016/j.yjmcc.2007.09.012 18061611

[43] Ibrahim AG, Cheng K, Marban E. (2014) Exosomes as critical agents of cardiac regeneration triggered by cell therapy. Stem cell reports 2:606–619. 10.1016/j.stemcr.2014.04.006 24936449 PMC4050492

[44] Fasanaro P, D'Alessandra Y, Magenta A, Pompilio G, Capogrossi MC. (2015) Micrornas: Promising biomarkers and therapeutic targets of acute myocardial ischemia. Current vascular pharmacology. 13(3):305–15. 10.2174/15701611113119990011 23713865

[45] Tseliou E, de Couto G, Terrovitis J, Sun B, Weixin L, Marban L et al (2014) Angiogenesis, cardiomyocyte proliferation and anti-fibrotic effects underlie structural preservation post-infarction by intramyocardially-injected cardiospheres. PLoS One 9:e88590 10.1371/journal.pone.0088590 24558402 PMC3928273

[46] Li TS, Cheng K, Lee ST, Matsushita S, Davis D, Malliaras K et al Cardiospheres recapitulate a niche-like microenvironment rich in stemness and cell-matrix interactions, rationalizing their enhanced functional potency for myocardial repair. Stem cells 28(11):2088–98. 10.1002/stem.532 20882531 PMC3405979

[47] Malliaras K, Ibrahim A, Tseliou E, Liu W, Sun B, Middleton RC et al (2014) Stimulation of endogenous cardioblasts by exogenous cell therapy after myocardial infarction. EMBO molecular medicine 6:760–777. 10.1002/emmm.201303626 24797668 PMC4203354

[48] Tseliou E, Reich H, de Couto G, Terrovitis J, Sun B, Liu W et al (2014) Cardiospheres reverse adverse remodeling in chronic rat myocardial infarction: Roles of soluble endoglin and tgf-beta signaling. Basic Res Cardiol 109:443 10.1007/s00395-014-0443-8 25245471

[49] Aminzadeh MA, Tseliou E, Sun B, Cheng K, Malliaras K, Makkar RR et al Therapeutic efficacy of cardiosphere-derived cells in a transgenic mouse model of non-ischaemic dilated cardiomyopathy. Eur Heart J 36(12):751–62. 10.1093/eurheartj/ehu196 24866210 PMC4368856

