# Supplementary figures and images for: Widespread Myocardial Delivery of Heart-Derived Stem Cells by Nonocclusive Triple-Vessel Intracoronary Infusion in Porcine Ischemic Cardiomyopathy: Superior Attenuation of Adverse Remodeling Documented by Magnetic Resonance Imaging and Histology
Source: PLoS One. 2016 Jan 19;11(1):e0144523. doi: 10.1371/journal.pone.0144523 (PMC4718597; doi:10.1371/journal.pone.0144523)

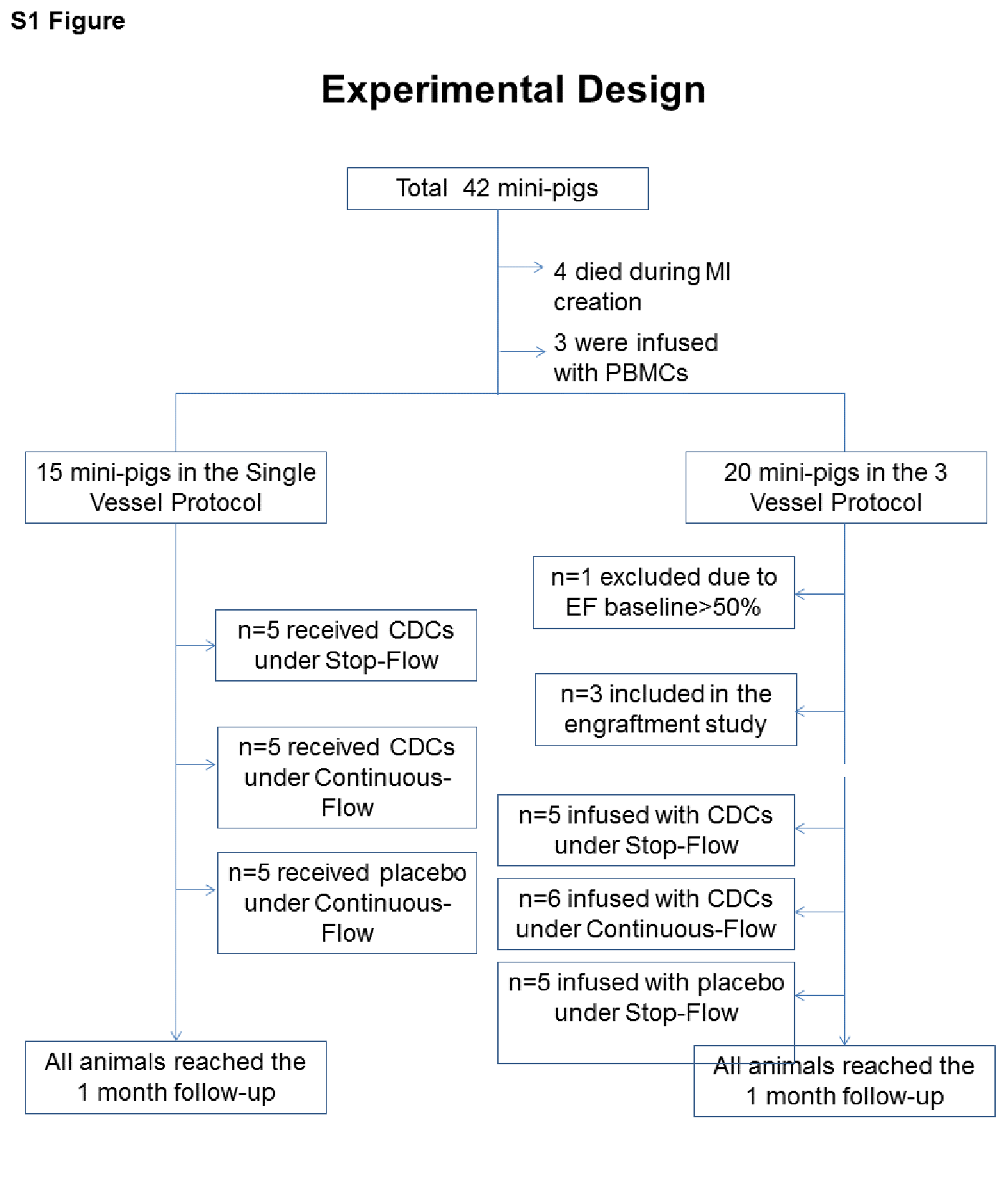

Supplement: S1 Fig — A total of 42 Yucatan mini-pigs were used in the current study including 2 substudies: n = 15 animals were infused only in the LAD and n = 19 animals were infused in all three coronary vessels. (TIF) [file pone.0144523.s001.tif]

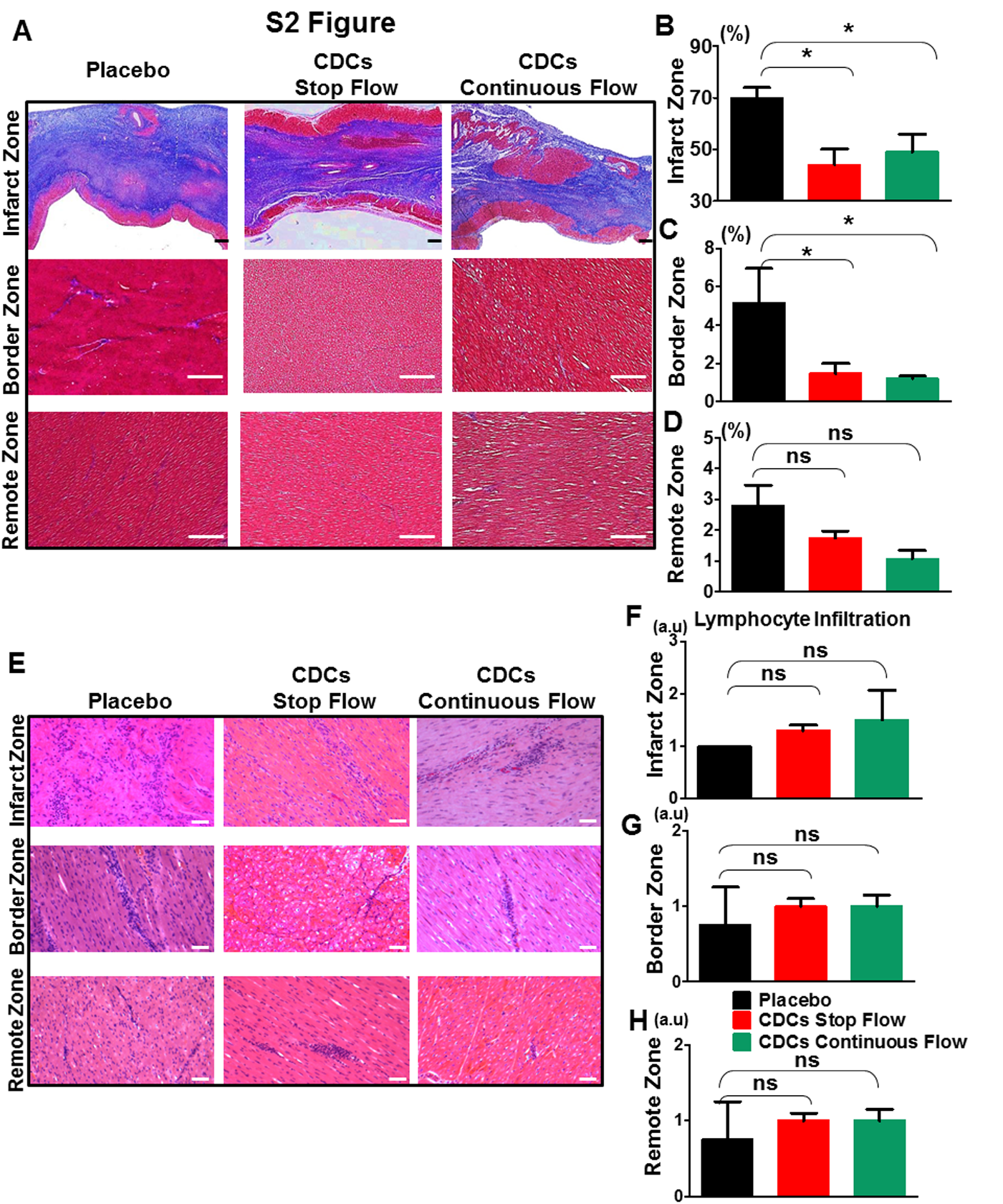

Supplement: S2 Fig — A. Representative Masson’s trichrome stained sections. B. The fibrotic tissue was significantly attenuated within the IZ and C., the BZ but not D., the RZ in both treated groups compared to the placebo. E. Representative hematoxylin & eosin stained sections. F., G., H. No significant mononuclear infiltration was observed 1 month post cell infusion compared to placebo. Error bars indicate SEM. Abbreviations: IZ, infarct zone; BZ, border zone; RZ, remote zone. Scale bar = 50μm. * p<0.05. (TIF) [file pone.0144523.s002.tif]

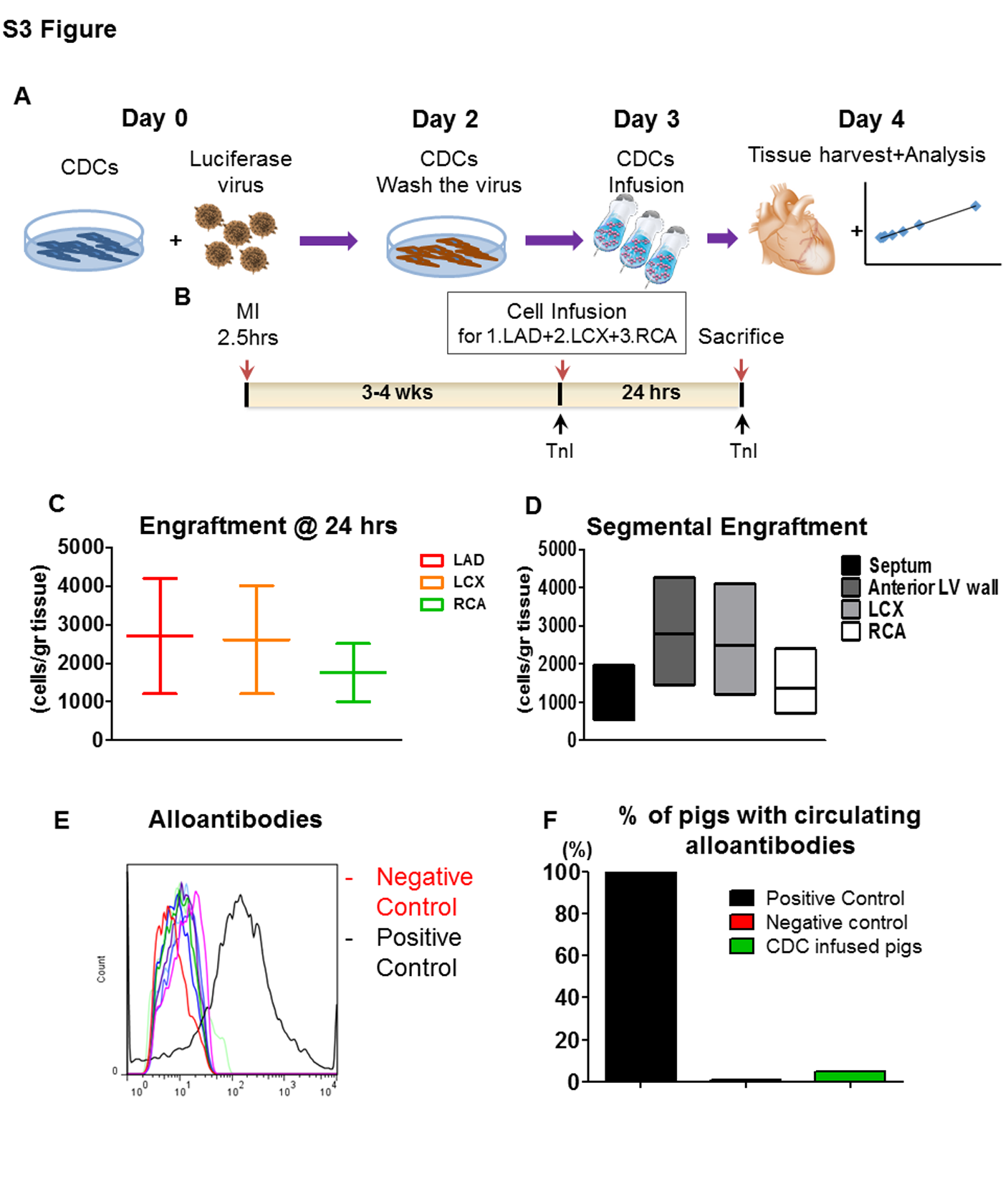

Supplement: S3 Fig — A. Scheme of the transduction and B. The timeline of the engraftment experiment. 12.5M CDCs were infused sequentially in each of the three coronary vessels. C. Strong Luc+ expression was found in the LAD and the LCX territories 24hrs post intracoronary infusion. Somewhat fewer cells engrafted in the RCA territory, but the differences among groups are not significant. D. Careful segmental analysis revealed that the anterior infarcted wall and the LCX territory received the majority of the Luc+ cells. E., F. Minimal detection of circulating alloantibodies post CDC infusion but the experiment is limited due to the lack of antibody quantification pre infusion. On the left plot, the red curve defines the negative control sample, the black curve the positive control and all the rest curves are the pig samples tested. Error bars indicate SEM. (TIF) [file pone.0144523.s003.tif]
